# Prevalence and genotypes of anal human papillomavirus infection among HIV-positive *vs*. HIV-negative men in Taizhou, China

**DOI:** 10.1017/S0950268818003205

**Published:** 2019-03-08

**Authors:** X. Liu, H. Lin, X. Chen, W. Shen, X. Ye, Y. Lin, Z. Lin, S. Zhou, M. Gao, Y. Ding, N. He

**Affiliations:** 1 Department of Epidemiology, School of Public Health, and the Key Laboratory of Public Health Safety of Ministry of Education, Fudan University, Shanghai, China; 2 Taizhou City Center for Disease Control and Prevention, Zhejiang Province, China; 3 Linhai District Center for Disease Control and Prevention, Zhejiang Province, China; 4 Sanmen District Center for Disease Control and Prevention, Zhejiang Province, China; 5 Wenlin District Center for Disease Control and Prevention, Zhejiang Province, China

**Keywords:** Anal infection, China, HIV/AIDS, HPV, male

## Abstract

This study aims to investigate the prevalence and genotype distribution of anal human papillomavirus (HPV) infection among men with different sexual orientations with or without human immunodeficiency virus (HIV) in China. A cross-sectional study was conducted during 2016–2017 in Taizhou City, Zhejiang Province. Convenient sampling was used to recruit male participants from HIV voluntary counselling and testing clinics and Center for Disease Control and Prevention. A face-to-face questionnaire interview was administered and an anal-canal swab was collected for HPV genotyping. A total of 160 HIV-positive and 113 HIV-negative men participated in the study. The prevalence of any type HPV was 30.6% for heterosexual men, 74.1% for homosexual and 63.6% for bisexual men among HIV-positive participants, while the prevalence was 8.3%, 29.2% and 23.8% respectively among HIV-negatives. The most prevalent genotypes were HPV-58 (16.9%), HPV-6 (15.6%) and HPV-11 (15.0%) among HIV-positive men, and were HPV-16 (4.4%), HPV-52 (4.4%) and HPV-6 (3.5%) among HIV-negative men. Having ever had haemorrhoids and having ever seen blood on tissue after defaecation was associated with HPV infection. One-fourth of the HPV infections in this study population can be covered by the quadrivalent vaccine in market. The highly prevalent anal HPV infection among men especially HIV-infected men calls for close observation and further investigation for anal cancer prevention.

## Introduction

Human papillomavirus (HPV) infection is the most common sexually transmitted infection (STI) worldwide. Mucosal HPV types from alpha genus infect squamous stratified epithelium from mucosal tissues, and some infections may persist for many years with the potential to cause cancers of cervix, vulva, vagina, penis, oropharynx, anus and rectum [1, 2]. The incidence of cervical cancer ranks fourth in women worldwide, with 85% of new cases occurred in less developed countries [3]. Anal squamous cell cancer, biologically similar to cervical cancer, is rare, but its incidence is increasing [4, 5]. Almost all cervical cancer and more than 80% of anal cancer are attributable to high-risk HPV (HR-HPV) infection including HPV types 16, 18, 31, 33, 35, 39, 45, 51, 52, 56, 58, 59, 68, 73 and 82 [6], and are mainly associated with HPV-16 and HPV-18 [7, 8]. These high-risk genotypes of HPV distribute worldwide with different prevalence in both women and men with lesions or abnormal cytology [9, 10]. The prevalence of HPV infection in Han women with cervical intraepithelial lesions 1, 2 and 3 and squamous cell carcinoma were over 90%, and were 10%–20% in general female Chinese population [11–13].

People living with human immunodeficiency virus (HIV) or having high-risk sexual behaviours including men who have sex with men (MSM) are at higher risk for HPV infection and HPV-related cancers [5, 14–16]. A meta-analysis of 53 studies mainly conducted in western countries reported that compared with HIV-negative MSM, HIV-positive MSM had higher prevalence of anal HPV infection (92.6% *vs.* 63.9%), higher prevalence of HR-HPV infection (73.5% *vs.* 37.2%) and higher prevalence of anal intraepithelial neoplasia (29.1% *vs.* 21.5%) [9]. The meta-analysis for studies in Chinese population conducted by our group reported a pooled prevalence of 71.7% for anal HPV infection and 50.7% for HR-HPV infection among HIV-positive MSM based on studies conducted in metropolises including Beijing, Chengdu and Shenzhen [17]. However, there lacks estimates for anal HPV prevalence among MSM from rural areas, where women had disproportionately higher burden from cervical cancer mortality [18]. And there is limited data for HPV infection among heterosexual men with high-risk sexual behaviours, despite the fact that most women get infected through intercourse with men. Moreover, the examination for potential correlates for HPV infection in the existing literature mainly focused on sexual behaviours [19–25]. Given the fact that there is no screening programme for anal cancer or HPV infection among male, local symptoms or history of anal disease might serve as indicator for further medical examination and care if any is found to be associated with HPV infection.

The HPV vaccines have been shown to be effective in preventing HPV infection and HPV related low- and high-grade squamous intraepithelial lesions and cancers among both women and men in western countries [26–28], and the nonavalent HPV vaccine have been shown to have significant increased potential impact compared with the quadrivalent vaccine [29, 30]. To what extent the vaccine can prevent HPV infection in Chinese men remains unclear. We conducted this cross-sectional study in Taizhou, a relatively rural area located on east coast of China to estimate the prevalence of anal HPV infection and genotype distribution among men of different sexual orientation with or without HIV infection, and to explore potential indicators for the existence of HPV infection.

## Methods

### Study design and subject

A cross-sectional study was conducted in Taizhou City, Zhejiang Province from August 2016 to October 2017. Participants were recruited by Taizhou City Center for Disease Control and Prevention (CDC) and three county-level CDCs. Convenient sampling was used to enrol HIV-positive men who were routinely followed-up by the four CDCs and HIV-negative men attending HIV Voluntary Counselling and Testing (VCT) clinics in the same CDCs. A total of 1286 HIV infected men had been routinely followed-up and 473 men had visited the VCT clinics during the study period. This study was approved by the Institutional Review Board of Fudan University (IRB#2016-TYSQ-06-2) and written informed consent was obtained from each participant.

### Data collection

Participants were face-to-face interviewed by trained health staff during the visit, and a questionnaire was used to ascertain epidemiological data. Socio-demographic characteristics including age, level of education, occupation, marital status with women, etc. were collected. Participants were also asked about their behavioural characteristics including sexual orientation, sexual behaviours, tobacco and alcohol use, history of illicit drug use, etc. Ever smoked was defined as having ever smoked more than 100 cigarettes lifetime. Frequency of alcohol drinking in the past year was recorded. Ever used illicit drugs was defined as having ever used any of the following substances: methamphetamine, ecstasy, heroin, marijuana, opium, cocaine, sedatives, ketamine or poppers (amyl nitrites). History of diagnosis of STIs including gonorrhoea, syphilis, chlamydia, condyloma acuminata and genital herpes was reported. Local symptoms of anus and history of haemorrhoids or anal fistula were collected. For HIV-infected participants, the most recent CD4^+^ T cell count was recorded, which was measured by flow cytometry (Becton, Dickinson and Company, USA) according to the manufacturer's protocol.

### Sample collection and laboratory tests

For each participant, an anal canal swab was collected by the trained staff using a nylon flocked swab. The staff inserted the swab into the anal canal for 2–3 cm, rotated it with gentle pressure against the canal wall while gradually moving it out. The whole process of swab sample collection was required to be no less than 2 min to gain enough cells. Then the swab was kept in a tube with 3 ml liquid medium using PBS as the main component to preserve the cells and stored at −20 °C for HPV DNA test.

HPV genotyping was performed using the HPV GenoArray Test Kit (Hybribio Ltd., Guangdong, China). The test kit detects 21 HPV genotypes using an L1 consensus primer-based polymerase chain reaction assay by the flow-through hybridisation technique with a TC-96/G/H^6^ HPV DNA Amplification Analyzer and an HMM-2 fast nucleic acid molecule hybridisation instrument. Among the 21 HPV genotypes, 15 are HR-HPV including 16, 18, 31, 33, 35, 39, 45, 51, 52, 53, 56, 58, 59, 66 and 68, and the other six are low-risk HPV (LR-HPV) including 6, 11, 42, 43, 44 and 81. Biotin control, internal control (IC), positive control and negative control from the kit were used in each laboratory test as quality control, and only when both of the biotin control and IC showed coloration, the test was defined as successful and the genotyping results could be read and reported.

### Statistical analyses

Statistical analyses were performed using SAS 9.3 statistical software (SAS Institute Inc., Cary, NC). *χ*^2^ test and Fisher exact test were used to compare the distribution of categorical variables where appropriate. Unconditional simple and multiple logistic regression were used to estimate the odds ratios (OR) and 95% confidence intervals (CI) in comparing prevalence of HPV infection across levels of covariates. A two-sided *P*-value <0.05 was considered as statistically significant.

## Results

### Socio-demographic characteristics

A total of 160 HIV-positive and 113 HIV-negative male participants were included in the study. The mean age was 42.3 years (s.d. 12.7) for HIV-positives and 31.2 years (s.d. 10.2) for HIV-negatives (*P* < 0.001). Level of education differed between the two groups (*P* < 0.001), with 9.9% of the HIV-positive participants received college education or above, and the proportion was 47.8% for HIV-negatives (Table 1).

**Table 1.** Socio-demographic characteristics and prevalence of anal HPV infection among HIV-negative *vs*. HIV-positive males in Taizhou, China, 2016–2017

	HIV negative						HIV positive					
	Overall		Any HPV positive		Any HR-HPV positive		Overall		Any HPV positive		Any HR-HPV positive	
	Number	Proportion %	Number	Prevalence %	Number	Prevalence %	Number	Proportion %	Number	Prevalence %	Number	Prevalence %
Total	113		24	21.2	17	15.0	160		82	51.2	75	46.9
Age (years)				*P* = 0.279		*P* = 0.527				*P* = 0.062		***P* = 0.046**
18–24	40	35.4	10	25.0	5	12.5	11	6.9	8	72.7	8	72.7
25–34	39	34.5	5	12.8	5	12.8	38	23.8	23	60.5	21	55.3
35–44	20	17.7	4	20.0	3	15.0	43	26.9	24	55.8	22	51.2
45–71	14	12.4	5	35.7	4	28.6	68	42.5	27	39.7	24	35.3
Level of education				*P* = 0.329		*P* = 0.163				***P* = 0.007**		***P* = 0.019**
Illiterate or primary school	10	8.9	3	30.0	2	20.0	39	25.7	11	28.2	10	25.6
Middle school	21	18.6	7	33.3	6	28.6	69	45.4	42	60.9	38	55.1
High school	28	24.8	5	17.9	4	14.3	29	19.1	18	62.1	16	55.2
College or above	54	47.8	9	16.7	5	9.3	15	9.9	8	53.3	8	53.3
Occupation				*P* = 0.533		*P* = 0.281				*P* = 0.589		*P* = 0.373
Student	7	6.7	1	14.3	0	0.0	3	1.9	3	100.0	3	100.0
Farmer	11	10.6	4	36.4	3	27.3	72	46.5	34	47.2	31	43.1
Business	38	36.5	5	13.2	3	7.9	38	24.5	21	55.3	20	52.6
Worker	20	19.2	5	25.0	3	15.0	18	11.6	8	44.4	8	44.4
Government	1	1.0	0	0.0	0	0.0	0	0.0	0	0.0	0	0.0
Jobless	7	6.7	2	28.6	2	28.6	10	6.5	6	60.0	6	60.0
Other	20	19.2	6	30.0	5	25.0	14	9.0	7	50.0	5	35.7
Sexual orientation				*P* = 0.075		*P* = 0.465				***P* < 0.001**		***P* < 0.001**
Hetero-sexual	36	32.4	3	8.3	3	8.3	72	46.2	22	30.6	20	27.8
Homo-sexual	48	43.2	14	29.2	9	18.7	58	37.2	43	74.1	39	67.2
Bi-sexual	21	18.9	5	23.8	4	19.0	22	14.1	14	63.6	13	59.1
Uncertain	6	5.4	2	33.3	1	16.7	4	2.6	2	50.0	2	50.0
Marital status with women				*P* = 0.680		*P* = 0.553				*P* = 0.235		*P* = 0.312
Single	69	62.7	14	20.3	11	15.9	54	35.1	33	61.1	30	55.6
Married	35	31.8	8	22.9	6	17.1	76	49.4	35	46.1	32	42.1
Divorced/widow	6	5.5	2	33.3	0	0.0	24	15.6	13	54.2	12	50.0
HR-HPV: high-risk HPV												

* *P*-value less than 0.05 was shown in bold.

### Sexual orientation

Among HIV-positive participants, 46.2% reported their sexual orientation as heterosexual, 37.2% homosexual, 14.1% bisexual and 2.6% uncertain. The sexual orientations reported by HIV-negative participants were 32.4% heterosexual, 43.2% homosexual, 18.9% bisexual and 5.4% uncertain (*P* = 0.114).

### Prevalence of HPV infection

Overall, the prevalence of any type of HPV infection was 51.2% among HIV-positive participants, and was 21.2% among HIV-negative men (*P* < 0.001). The prevalence of any HR-HPV was 46.9% among HIV-positive participants and 15.0% among HIV-negative men (*P* < 0.001) (Table 1).

### Genotypes of HPV infection

**Fig. 1.**
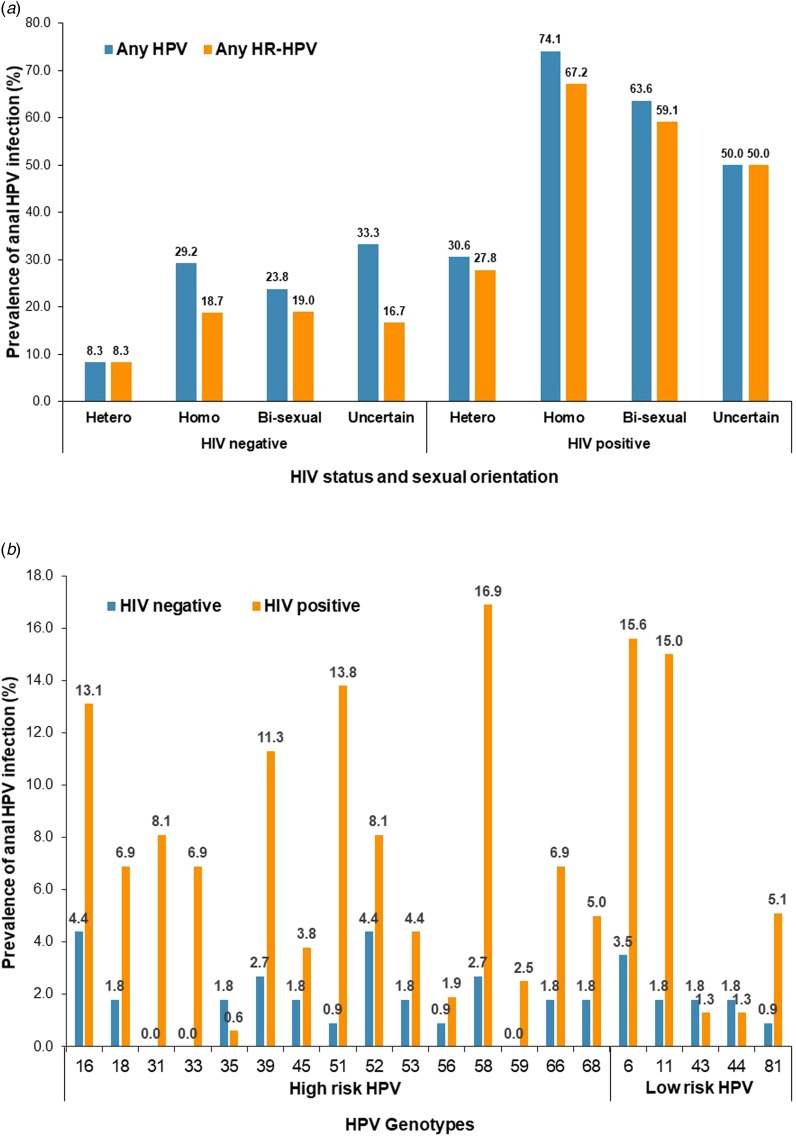
Prevalence of anal HPV infection by HIV status, sexual orientation and HPV genotype.

The most prevalent genotypes were HPV-58 (16.9%), HPV-6 (15.6%), HPV-11 (15.0%), HPV-51 (13.8%), HPV-16 (13.1%) and HPV-39 (11.3%) among HIV-positive participants, and were HPV-16 (4.4%), HPV-52 (4.4%), HPV-6 (3.5%), HPV-39 (2.7%) and HPV-58 (2.7%) among HIV-negative participants (Fig. 1).

Thirty-seven percent of the HIV-positive participants were infected by two or more than two types of HPV, while the prevalence of multiple infection was 6.2% for HIV-negative men (*P* < 0.001).

### Prevalence of HPV infection by HIV status and sexual orientation

For HIV-positive participants, the prevalence of any type of HPV infection were 30.6% for heterosexual men, 74.1% for homosexual, 63.6% for bisexual and 50.0% for those with uncertainty (*P* < 0.001), and the prevalence of any type of HR-HPV infection was 27.8%, 67.2%, 59.1% and 50.0%, respectively (*P* < 0.001) (Table 1 and Fig. 1).

For HIV-negative participants, the prevalence of any HPV infection was 8.3% for heterosexual men, 29.2% for homosexual, 23.8% for bisexual and 33.3% for those with uncertainty (*P* = 0.075), and the prevalence of any HR-HPV infection was 8.3%, 18.7%, 19.0% and 16.7%, respectively (*P* = 0.465) (Table 1 and Fig. 1).

### Risk factors for HPV infection

Among HIV-positive participants, the prevalence of anal HPV infection decreased with the increase of age, and the association was statistically significant for HR-HPV infection. The prevalence of HR-HPV was 72.7% among those under 25 years old, and was 35.3% among those over 45 years (*P* = 0.046) (Table 1).

In simple logistic regression models for HIV-positive participants, having ever smoked (OR 0.40, 95% CI 0.21–0.76) and having ever had heterosexual behaviour (OR 0.22, 95% CI 0.11–0.45) were inversely associated with any type HPV infection; while having drunk alcohol once per month or less in the past year (OR 3.00, 95% CI 1.08–8.35), having ever seen blood on tissue after defaecation (OR 2.42, 95% CI 1.22–4.78), having ever had homosexual behaviour (OR 5.36, 95% CI 2.70–10.65) were positively associated with HPV infection. Similar associations were observed for any HR-HPV infection (Table 2).

**Table 2.** Simple logistic regression analyses of correlates of anal HPV infection among HIV-negative *vs*. HIV-positive males in Taizhou, China, 2016–2017

	HIV negative				HIV positive			
Characteristics	Number	%	Any HPV	Any HR-HPV	Number	%	Any HPV	Any HR-HPV
Overall	113				160			
Ever smoked *vs*. never	27	23.9	1.42 (0.52–3.91)	1.95 (0.64–5.89)	66	41.3	**0.40 (0.21–0.76)**	**0.43 (0.23–0.83)**
Drank ⩽once/month in the past year *vs*. no drink	30	26.5	2.75 (0.95–7.96)	3.09 (0.91–10.44)	25	15.6	**3.00 (1.08–8.35)**	2.57 (0.96–6.86)
Drank >once/month in the past year *vs*. no drink	40	35.4	0.46 (0.13–1.66)	0.58 (0.13–2.63)	59	36.9	0.65 (0.33–1.29)	**0.47 (0.23–0.96)**
Ever used drug *vs*. never	12	10.6	**4.61 (1.33–15.95)**	3.38 (0.89–12.85)	3	1.9	1.93 (0.17–21.66)	2.30 (0.20–25.90)
History of STI in the past year *vs*. no STI	7	6.2	3.04 (0.63–14.61)	0.94 (0.11–8.32)	9	5.6	1.97 (0.48–8.18)	2.38 (0.57–9.86)
Ever seen blood after defaecation *vs*. never	68	60.2	2.16 (0.78–5.98)	2.24 (0.68–7.41)	101	63.1	**2.42 (1.22–4.78)**	**2.32 (1.17–4.64)**
Ever had haemorrhoids *vs*. never	50	44.2	**2.57 (1.02–6.51)**	**3.66 (1.19–11.23)**	34	21.3	1.09 (0.51–2.33)	1.17 (0.55–2.50)
Had homo-sexual behaviour *vs*. never	75	66.4	**4.28 (1.18–15.46)**	2.52 (0.68–9.42)	84	52.5	**5.36 (2.70–10.65)**	**4.68 (2.37–9.26)**
Age at start homo-sex <23 years old *vs*. older	35	46.7	1.27 (0.45–3.62)	1.38 (0.42–4.51)	33	39.3	0.74 (0.27–2.04)	0.79 (0.31–2.04)
Number of lifetime homo-sex partners >5 *vs*. less	23	30.7	**3.76 (1.29–10.97)**	1.90 (0.57–6.29)	25	29.8	0.95 (0.33–2.75)	0.86 (0.32–2.35)
Had anal sex in the past 6 months *vs*. did not	65	86.7	4.00 (0.47–33.73)	2.25 (0.26–19.38)	51	60.7	1.32 (0.51–3.42)	1.30 (0.52–3.23)
Had receptive intercourse in the past 6 months *vs*. did not	43	57.3	**3.92 (1.00–15.39)**	7.74 (0.93–64.24)	43	51.2	3.30 (0.70–15.64)	2.31 (0.50–10.67)
Did not use condom for the last homo-sex *vs*. used	29	38.7	2.20 (0.79–6.14)	1.77 (0.55–5.72)	47	56.0	0.62 (0.24–1.63)	0.62 (0.25–1.56)
Had casual homo-sex partner in past 6 month *vs*. did not	23	30.7	2.14 (0.75–6.17)	1.94 (0.59–6.43)	13	15.5	0.43 (0.13–1.43)	**0.28 (0.08–0.96)**
Had hetero-sexual behaviour *vs*. never	63	55.8	0.46 (0.18–1.15)	0.63 (0.22–1.78)	98	61.3	**0.22 (0.11–0.45)**	**0.27 (0.14–0.54)**
Number of lifetime hetero-sex partners >2 *vs*. less	34	54.0	0.51 (0.13–2.03)	0.83 (0.19–3.68)	72	73.5	0.40 (0.16–1.02)	0.41 (0.16–1.06)
Did not use condom for the last hetero-sex *vs*. used	43	68.3	2.06 (0.40–10.72)	1.46 (0.27–7.96)	58	59.2	0.77 (0.34–1.75)	0.88 (0.38–2.03)
Most recent CD4+ T cell counts <500 cells/μl *vs*. more					91	56.9	0.64 (0.34–1.21)	0.72 (0.38–1.35)

* Confidence intervals with *P*-value less than 0.05 were shown in bold.

For HIV-negative participants, having ever used illicit drugs (OR 4.61, 95% CI 1.33–15.95), having ever had haemorrhoids (OR 2.57, 95% CI 1.02–6.51), having ever had homo sex (OR 4.28, 95% CI 1.18–15.46) were significantly associated with any type HPV infection. Having more than five lifetime homo-sex partners (OR 3.76, 95% CI 1.29–10.97) and having had receptive intercourse in the past six months (OR 3.92, 95% CI 1.00–15.39) were also associated with HPV infection. Only having ever had haemorrhoids (OR 3.66, 95% CI 1.19–11.23) was associated with any HR-HPV infection among HIV negative participants (Table 2).

Since sexual behaviours correlate with each other, having ever had homosexual behaviour was chosen as the indicator for sexual behaviours and was put into the multiple logistic regression model together with age, smoking status and drinking frequency. For HIV-positive participants, having drunk alcohol once or less per month in the past year (OR 3.64, 95% CI 1.18–11.20) compared with those did not drink, and having ever had homosexual behaviour (OR 4.74, 95% CI 2.25–9.97) were significantly associated with any type HPV infection, and the latter was also significantly associated with HR-HPV infection (OR 3.87, 95% CI 1.85–8.11). For HIV-negative participants, having ever had homosexual behaviour was significantly associated with risk of any type of HPV infection (OR 4.36, 95% CI 1.13–16.86) (Table 3).

**Table 3.** Multiple logistic regression analyses of correlates of anal HPV infection among HIV-negative *vs*. HIV-positive males in Taizhou, China, 2016–2017

Characteristics	HIV-negative		HIV-positive	
	Any HPV	Any HR-HPV	Any HPV	Any HR-HPV
Age (continuous)	1.01 (0.96–1.06)	1.03 (0.98–1.09)	0.99 (0.96–1.02)	0.99 (0.96–1.02)
Ever smoked				
No	1.00	1.00	1.00	1.00
Yes	1.59 (0.52–4.88)	2.01 (0.61–6.62)	0.54 (0.25–1.17)	0.67 (0.31–1.45)
Drank alcohol in the past year				
Never	1.00	1.00	1.00	1.00
Drink <once/month	2.37 (0.78–7.14)	2.66 (0.76–9.34)	**3.64 (1.18–11.20)**	2.79 (0.96–8.16)
Drink >once/month	0.43 (0.11–1.62)	0.55 (0.12–2.55)	1.00 (0.45–2.24)	0.64 (0.29–1.43)
Had homo-sexual behaviour				
No	1.00	1.00	1.00	1.00
Yes	**4.36 (1.13–16.86)**	2.49 (0.62–10.01)	**4.74 (2.25–9.97)**	**3.87 (1.85–8.11)**
Adjusted for all other variables in the table.				

* Confidence intervals with *P*-value less than 0.05 were shown in bold.

### Coverage of the HPV infection by vaccines

Overall, the bivalent vaccine (HPV-16 and HPV-18) covers 13.5% of HPV genotypes infected by the study participants, the quadrivalent vaccine (bivalent plus HPV-6 and HPV-11) covers 24.5%, while the nine-valent (quadrivalent plus HPV-31, 33, 45, 52 and 58) covers 31.4%.

## Discussion

This cross-sectional study reported prevalence of anal HPV infection, genotype distribution and the factors associated with the infection among men with or without HIV from Taizhou, a relatively rural area in Zhejiang from east-coast China. As far as we know, this was the first study to directly compare HPV prevalence among male with different sexual orientations by HIV status in the Chinese population.

The prevalence of anal HPV infection was 51.2% among HIV-positive male, more than double the prevalence of 21.2% among HIV-negatives in our study population. Similar to the observations from former studies, HIV infection highly correlates with HPV infection [9, 17]. Research conducted in several Chinese cities including Beijing, Xi'an, Chengdu and Shenzhen etc. reported prevalence of any HPV type ranging from 45.3% to 99.0% among HIV-positive MSM, higher than the prevalence for HIV-negative MSM (33.8%–62.8%) [19–25]. The anal HPV prevalence of 74.1% for HIV-positive MSM in our study was in high concordance with the pooled prevalence in Chinese MSM [17]. HIV and HPV have shared transmission routes including high-risk sexual behaviours. MSM tended to have more anal sex and more sex partners, led to the higher possibility of viral transmission [31]. The role played by risky behaviours was also confirmed by our observation that homosexual or bisexual men showed higher prevalence of anal HPV infection than heterosexual men in both HIV positive and negative participants.

In addition to the shared risk factors, other explanations existed for the high correlation between HIV and HPV infection. The lesion caused by HPV infection might facilitate the transmission of HIV, and the immune depression caused by HIV might hamper the ability in clearing HPV infection [32–34]. However, the association between CD4+ T cell counts and HPV infection remained controversial [35–39]. And our study did not observe a clear link between recent CD4 and HPV prevalence. The cross-sectional nature of the study limited the ability to draw conclusion, thus further prospective studies are needed to clarify the association and understand the natural history of anal HPV infection with or without the impact from HIV infection.

The most prevalent HR-HPV genotypes reported in this study were HPV-58, HPV-51, HPV-16 and HPV-39 for HIV-positive male and HPV-16, HPV-52, HPV-39 and HPV-58 for HIV-negatives. Meanwhile, the LR-HPVs including HPV-6 and HPV-11 which mainly cause genital warts were also highly prevalent in the study population. The spectrum of high-prevalent genotypes was different from those in western countries and had important implication in vaccine development [9]. For the current vaccines in market, the nine-valet one can cover 31.4% of the infections in this study population, while the quadrivalent one can cover 24.5%. More comprehensive studies on HPV genotype distributions and vaccine effectiveness in Chinese population are warranted.

Common anal symptoms and diseases were asked and two were found to be associated with HPV infection in the study population. For HIV-negative men, having ever had haemorrhoids showed positive association with HPV. For HIV-positive men, having ever seen blood on tissues after defaecation showed positive association. Common anal diseases and symptoms are important indicators for anal cancer, for existing anal conditions might increase the risk for the viral infection at local mucosa [15]. And the two discovered by our study might serve as indicators for people at higher risk for further screening and examination.

There were several limitations to the current study. First of all, the selection of the study participants was not random. Thus the reported prevalence might not be representative for all males with high-risk behaviours in Taizhou. Second, the cross-sectional nature of the study limited the conclusions we may draw for the associations observed. Third, the risk behaviours were measured based on self-report, which might lead to information bias. Fourth, although health professionals were trained using a standard protocol, the collection of anal-canal cell samples might not be completely successful, and the test kit for HPV included 21 genotypes, which might together lead to an underestimate of the prevalence.

Accumulatively 758 000 people were living with HIV/AIDS in China by the end of 2017. Among the newly diagnosed, more than 70% were male; 70% were transmitted by hetero-sexual contact and 26% were by homo-sex [40]. Given the size of the HIV population and the high prevalence of HPV infection in men with high-risk behaviours, we appeal for further investigations in incidence and progression of anal HPV infection in men, to decide whether a screening programme similar to the one for cervical cancer should be implemented to reduce the cancer risk and viral transmission.
